# Supplementary material for: Positional scanning and computational modeling reveal determinants of legumain transpeptidase activity
Source: Protein Sci. 2026 Apr 6;35(5):e70563. doi: 10.1002/pro.70563 (PMC13052201; doi:10.1002/pro.70563)
Supplement: Supplementary file 1 — FIGURE S1. Human legumain prefers positively charged amino acids at position P2" for efficient ligation. (a–d) MALDI‐ToF mass spectra of selected SFTI variants after incubation with human legumain (hLEG): (A) G1A2…FPN14GLA peptide, (b) G1H2…FG13N14GLA peptide, (c) G1A2…FG13N14GK16A peptide, and (d) G1H2…FG13N14GK16A peptide. The indicated peptides were resynthesized at >95% purity. Peaks corresponding to the unprocessed SFTI precursor, the cyclic SFTI product (C‐SFTI), and the linear intermediate lacking residues G15LA17 or G15KA17 (L‐SFTI) are annotated. FIGURE S2. Transpeptidation efficiency of legumain depends on its fold stability and substrate‐binding kinetics. (a) Human legumain was preincubated at pH 4.0 or pH 6.0 for 2 min at 37°C. Cyclization was subsequently assayed at pH 6.0 with 60 nM enzyme. The relative amount of cyclized SFTIopt (C‐SFTIopt) was expressed as the percentage of total SFTIopt in the reaction. (b) Eadie‐Hofstee transformation of human legumain‐catalyzed SFTIopt cyclization. Points represent experimental data; the solid line shows the fit to the allosteric sigmoidal model (Equation 1), and the dashed line shows the fit to the Michaelis–Menten model. The Eadie–Hofstee plot supports that the SFTI‐cyclisation reaction does not follow classical Michaelis–Menten kinetics. FIGURE S3. The S2' pocket is a key determinant of substrate specificity. (a, b) AlphaFold 3 models of human legumain bound to the indicated peptides: (a) FPN14‐GR, (B) FGN14‐GR. (c, d) AlphaFold 3 models of human legumain V155G‐D160Y bound to the indicated peptides: (c) FGN14‐GL, (d) FGN14‐GK. FIGURE S4. Legumain hydrolase activity analyzed based on the amount of linear product formed. Results of the positional peptide scanning assay using a crude peptide library of 140 variants are summarized in a heatmap. The relative amount of linear SFTI product (L‐SFTI) formed was normalized to the peptide containing alanine at the respective position. FIGURE S5. P1'‐Cys interacts with hum [file PRO-35-e70563-s001.pdf]

Supporting Information

Positional Scanning and Computational Modeling Reveal Determinants of Human Legumain Transpeptidase Activity

Rupert Klaushofer[1,2], Sven O. Dahms[1,2], Hans Brandstetter[1,2], Elfriede Dall[1,2,*]

Affiliations:

[1]Department of Biosciences and Medical Biology, University of Salzburg, 5020 Salzburg, Austria.

[2]Center of Tumor Biology and Immunology, University of Salzburg, 5020 Salzburg, Austria.

Corresponding Author:

[*]Elfriede Dall (elfriede.dall@plus.ac.at)

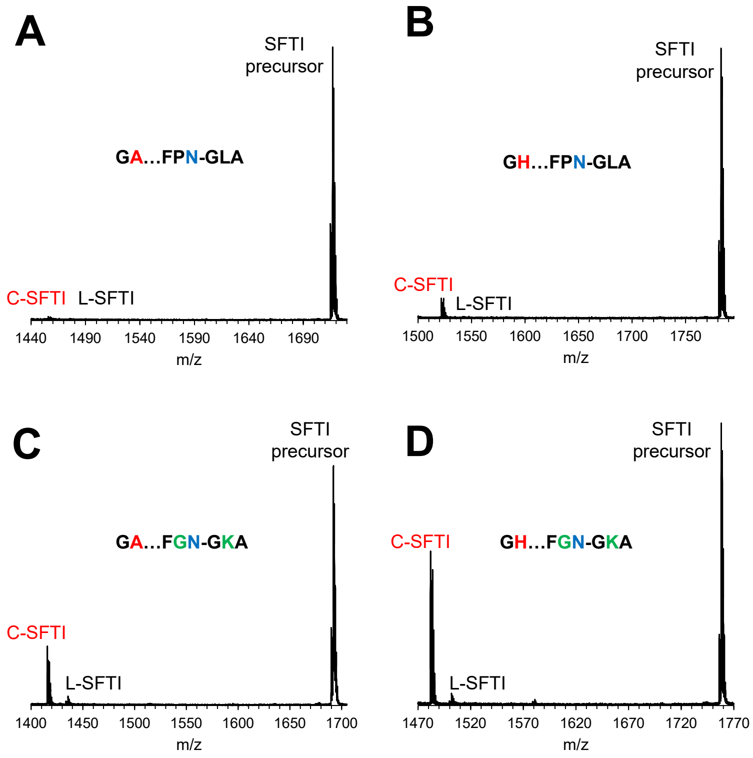

Supplementary Figure 1. Human legumain prefers positively charged amino acids at position P2" for efficient ligation. (A-D) MALDI-ToF mass spectra of selected SFTI variants after incubation with human legumain (hLEG): **(A)** $G^{1}A^{2}$…$FPN^{14}GLA$ peptide, **(B)** $G^{1}H^{2}$…$FG^{13}N^{14}GLA$ peptide, **(C)** $G^{1}A^{2}$…$FG^{13}N^{14}GK^{16}A$ peptide, and **(D)** $G^{1}H^{2}$…$FG^{13}N^{14}GK^{16}A$ peptide. The indicated peptides were resynthesized at >95% purity. Peaks corresponding to the unprocessed SFTI precursor, the cyclic SFTI product (C-SFTI), and the linear intermediate lacking residues $G^{15}LA^{17}$ or $G^{15}KA^{17}$ (L-SFTI) are annotated.

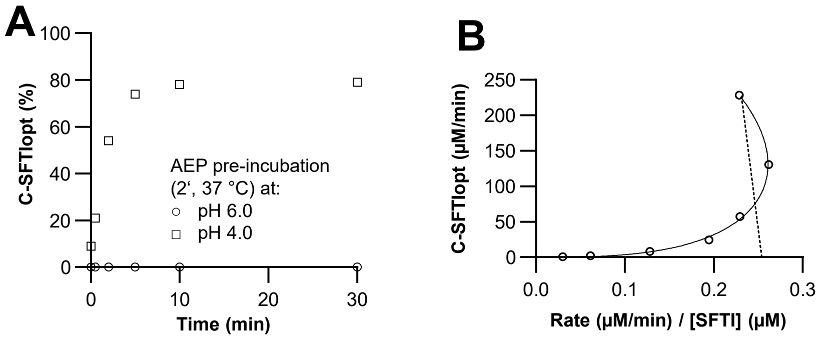

Supplementary Figure 2. Transpeptidation efficiency of legumain depends on its fold stability and substrate-binding kinetics. (A) Human legumain was preincubated at pH 4.0 or pH 6.0 for 2 min at 37 °C. Cyclization was subsequently assayed at pH 6.0 with 60 nM enzyme. The relative amount of cyclized $SFTI_{opt}$ (C-$SFTI_{opt}$) was expressed as the percentage of total $SFTI_{opt}$ in the reaction. **(B)** Eadie-Hofstee transformation of human legumain-catalyzed $SFTI_{opt}$ cyclization. Points represent experimental data; the solid line shows the fit to the allosteric sigmoidal model (Eq. 1), and the dashed line shows the fit to the Michaelis-Menten model. The Eadie-Hofstee plot supports that the SFTI-cyclisation reaction does not follow classical Michaelis-Menten kinetics.

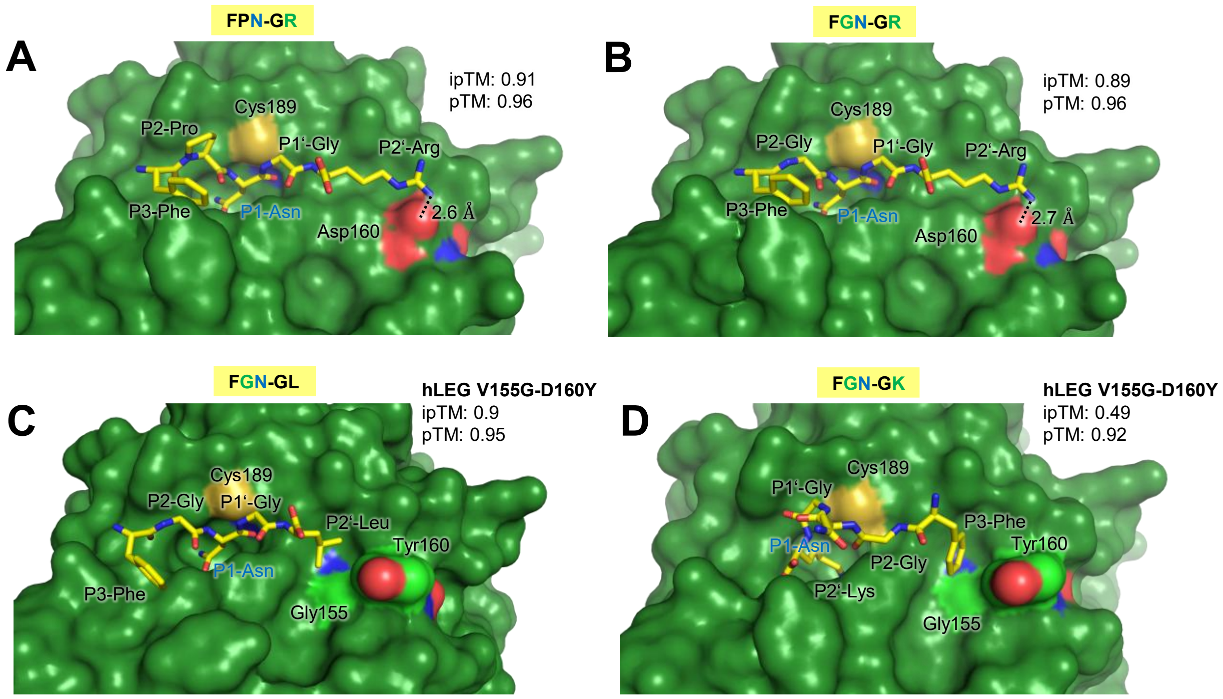

Supplementary Figure 3. The S2' pocket is a key determinant of substrate specificity. **(A,B)** AlphaFold 3 models of human legumain bound to the indicated peptides: **(A)** FPN^{14}–GR, **(B)** FGN^{14}–GR. **(C, D)** AlphaFold 3 models of human legumain V155G-D160Y bound to the indicated peptides: **(C)** FGN^{14}–GL, **(D)** FGN^{14}–GK.

Linear product (normalized, Ala = 1)							
	P1"	P2"	P3	P2	P1'	P2'	P3'
A	1.0	1.0	1.0	1.0	1.0	1.0	1.0
C	0.1	0.2	0.3	1.8	7.6	0.9	5.7
D	0.5	0.9	0.1	0.2	0.2	0.6	1.5
E	0.8	0.1	0.1	1.3	0.5	0.4	0.8
F	0.4	0.3	0.3	0.1	0.6	0.1	0.0
G	0.4	0.7	0.1	0.2	5.6	0.4	1.8
H	0.5	0.6	0.1	0.2	5.9	1.7	2.2
I	0.8	0.4	0.1	0.1	0.3	0.7	0.3
K	0.7	1.4	0.1	0.2	3.0	2.9	3.2
L	0.5	0.3	0.2	0.1	0.4	0.4	2.7
M	0.3	0.4	0.3	0.2	0.5	0.3	0.3
N	0.2	0.7	0.3	0.2	0.5	0.3	0.7
P	0.3	0.2	0.1	0.3	0.9	0.3	0.2
Q	0.0	0.1	0.1	0.6	0.7	0.6	0.4
R	0.9	0.7	0.9	1.4	1.7	2.3	0.9
S	0.2	0.1	0.7	1.0	17.0	0.8	1.1
T	0.2	0.1	0.2	0.2	0.7	1.3	2.3
V	0.8	0.1	0.2	0.7	1.3	0.2	0.3
W	0.0	0.1	0.1	0.2		0.0	0.3
Y	0.4	0.2	0.1	0.1	0.6	0.4	0.7

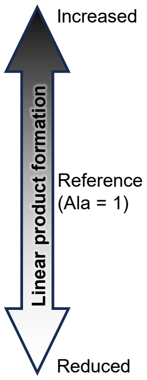

Supplementary Figure 4. Legumain hydrolase activity analyzed based on the amount of linear product formed. Results of the positional peptide scanning assay using a crude peptide library of 140 variants are summarized in a heatmap. The relative amount of linear SFTI product (L-SFTI) formed was normalized to the peptide containing alanine at the respective position.

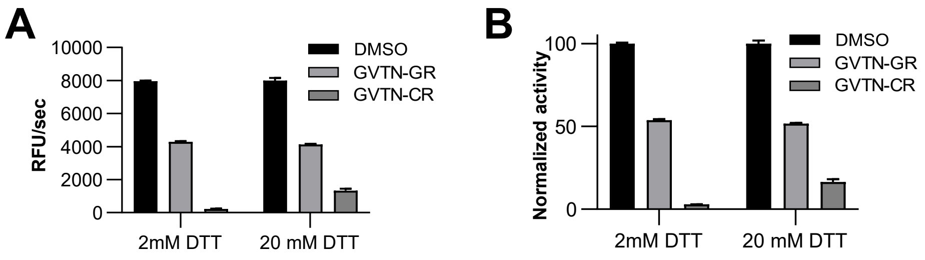

Supplementary Figure 5. P1′-Cys interacts with human legumain through disulfide bond formation. (A) Competition assays of human legumain with the peptides GVTN-CR and GVTN-GR. Each peptide (2 mM) was preincubated with the fluorogenic substrate Z-AAN-AMC in assay buffer (pH 5.5) containing either 2 mM or 20 mM DTT. Reactions were initiated by the addition of 2 nM human legumain, and fluorescence was monitored over 20 min at 37 °C. **(B)** Same experiment as in **(A)**, with data normalized to the respective DMSO control reactions.

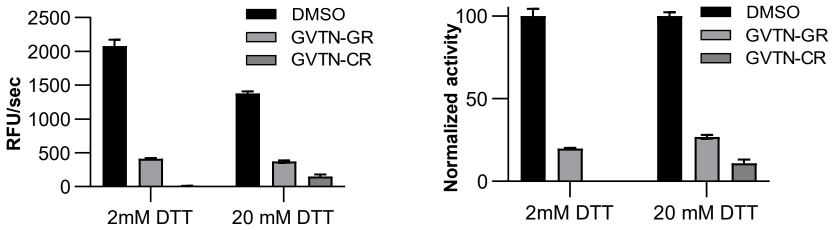

Supplementary Figure 6. P1′-Cys interacts with *Arabidopsis thaliana* legumain β (AtLEGβ) through disulfide bond formation. (A) Competition assays of AtLEGβ with the peptides GVTN-CR and GVTN-GR. Each peptide (2 mM) was preincubated with the fluorogenic substrate Z-AAN-AMC in assay buffer (pH 5.5) containing either 2 mM or 20 mM DTT. Reactions were initiated by the addition of 20 nM AtLEGβ, and fluorescence was monitored over 20 min at 37 °C. **(B**) Same experiment as in **(A)**, with data normalized to the respective DMSO control reactions.

A

AtLEGβ + AXXN-CL
(colored according to ipTM score)

P2 \ P3	A	C	D	E	F	G	H	I	K	L	M	N	P	Q	R	S	T	V	W	Y
**A**	0.94	0.91	0.93	0.94	0.92	0.93	0.94	0.94	0.93	0.93	0.94	0.94	0.94	0.94	0.93	0.93	0.95	0.95	0.94	0.93
**C**	0.94	0.91	0.93	0.94	0.93	0.93	0.94	0.94	0.93	0.93	0.94	0.94	0.94	0.94	0.93	0.94	0.95	0.94	0.93	0.92
**D**	0.94	0.87	0.93	0.94	0.92	0.93	0.93	0.94	0.93	0.94	0.93	0.94	0.93	0.94	0.94	0.93	0.95	0.95	0.94	0.93
**E**	0.94	0.90	0.92	0.94	0.92	0.93	0.93	0.95	0.92	0.93	0.92	0.93	0.93	0.94	0.93	0.93	0.95	0.95	0.93	0.93
**F**	0.94	0.82	0.93	0.93	0.92	0.92	0.92	0.93	0.91	0.92	0.91	0.93	0.92	0.93	0.93	0.92	0.94	0.94	0.93	0.92
**G**	0.92	0.87	0.93	0.94	0.93	0.90	0.94	0.94	0.92	0.92	0.93	0.93	0.93	0.93	0.93	0.92	0.94	0.95	0.93	0.93
**H**	0.94	0.88	0.93	0.94	0.92	0.93	0.94	0.94	0.93	0.93	0.92	0.94	0.93	0.93	0.93	0.93	0.94	0.94	0.93	0.93
**I**	0.94	0.82	0.94	0.94	0.93	0.94	0.92	0.94	0.92	0.92	0.93	0.93	0.92	0.94	0.93	0.93	0.95	0.95	0.93	0.92
**K**	0.94	0.91	0.94	0.94	0.92	0.94	0.94	0.94	0.93	0.93	0.93	0.94	0.94	0.93	0.94	0.93	0.95	0.95	0.94	0.93
**L**	0.94	0.87	0.93	0.93	0.91	0.94	0.92	0.93	0.92	0.92	0.93	0.94	0.92	0.94	0.93	0.93	0.94	0.95	0.93	0.92
**M**	0.94	0.91	0.93	0.94	0.93	0.94	0.94	0.94	0.93	0.93	0.93	0.94	0.94	0.94	0.94	0.93	0.95	0.95	0.93	0.92
**N**	0.94	0.91	0.93	0.94	0.92	0.93	0.94	0.94	0.93	0.93	0.94	0.94	0.93	0.94	0.94	0.93	0.95	0.95	0.93	0.93
**P**	0.94	0.90	0.94	0.94	0.93	0.89	0.94	0.94	0.93	0.93	0.94	0.93	0.94	0.94	0.93	0.93	0.95	0.95	0.94	0.94
**Q**	0.94	0.90	0.93	0.94	0.92	0.93	0.93	0.94	0.93	0.93	0.93	0.94	0.94	0.93	0.93	0.93	0.95	0.95	0.94	0.93
**R**	0.94	0.92	0.93	0.94	0.92	0.94	0.94	0.94	0.93	0.93	0.93	0.94	0.94	0.94	0.94	0.93	0.95	0.95	0.93	0.92
**S**	0.93	0.86	0.93	0.94	0.92	0.93	0.94	0.95	0.93	0.93	0.94	0.93	0.94	0.93	0.93	0.93	0.95	0.95	0.94	0.92
**T**	0.94	0.90	0.94	0.94	0.92	0.93	0.94	0.95	0.94	0.93	0.94	0.94	0.94	0.94	0.94	0.94	0.95	0.95	0.93	0.92
**V**	0.94	0.88	0.94	0.94	0.92	0.94	0.92	0.95	0.93	0.93	0.93	0.94	0.93	0.94	0.93	0.94	0.95	0.95	0.93	0.92
**W**	0.93	0.82	0.93	0.93	0.92	0.91	0.93	0.93	0.92	0.92	0.92	0.93	0.92	0.93	0.93	0.92	0.94	0.94	0.93	0.93
**Y**	0.94	0.86	0.92	0.93	0.92	0.93	0.93	0.93	0.93	0.92	0.92	0.93	0.92	0.93	0.93	0.92	0.93	0.94	0.92	0.92

High ipTM
ipTM (AtLEGβ + peptide)
Low ipTM

B

AtLEGβ + XXAN-CL
(colored according to ipTM score)

P3 \ P4	A	C	D	E	F	G	H	I	K	L	M	N	P	Q	R	S	T	V	W	Y
**A**	0.94	0.90	0.93	0.93	0.94	0.94	0.95	0.95	0.94	0.95	0.94	0.94	0.94	0.94	0.94	0.93	0.94	0.94	0.94	0.94
**C**	0.91	0.92	0.91	0.91	0.94	0.89	0.92	0.92	0.90	0.93	0.90	0.88	0.89	0.91	0.87	0.89	0.91	0.93	0.92	0.93
**D**	0.93	0.92	0.92	0.92	0.95	0.94	0.94	0.94	0.93	0.95	0.93	0.93	0.93	0.93	0.93	0.92	0.94	0.94	0.93	0.95
**E**	0.94	0.92	0.92	0.94	0.94	0.94	0.95	0.95	0.95	0.95	0.94	0.94	0.94	0.93	0.94	0.94	0.95	0.95	0.93	0.95
**F**	0.92	0.91	0.93	0.92	0.93	0.93	0.92	0.93	0.92	0.94	0.93	0.93	0.92	0.93	0.93	0.93	0.93	0.93	0.93	0.93
**G**	0.93	0.88	0.93	0.93	0.94	0.94	0.94	0.93	0.92	0.93	0.93	0.93	0.94	0.93	0.92	0.93	0.94	0.93	0.94	0.94
**H**	0.94	0.92	0.94	0.93	0.94	0.94	0.94	0.94	0.93	0.94	0.94	0.94	0.94	0.93	0.93	0.93	0.94	0.94	0.94	0.94
**I**	0.94	0.91	0.94	0.94	0.95	0.94	0.96	0.95	0.95	0.95	0.95	0.93	0.94	0.93	0.94	0.94	0.95	0.95	0.95	0.94
**K**	0.93	0.89	0.93	0.93	0.93	0.93	0.94	0.94	0.94	0.94	0.93	0.92	0.93	0.93	0.93	0.92	0.93	0.94	0.93	0.94
**L**	0.93	0.89	0.93	0.93	0.94	0.93	0.93	0.94	0.92	0.94	0.93	0.92	0.93	0.92	0.92	0.93	0.93	0.93	0.93	0.93
**M**	0.94	0.90	0.93	0.92	0.95	0.94	0.94	0.94	0.94	0.95	0.94	0.92	0.94	0.92	0.94	0.93	0.93	0.94	0.94	0.94
**N**	0.94	0.90	0.93	0.93	0.95	0.94	0.93	0.94	0.93	0.93	0.94	0.93	0.94	0.93	0.94	0.94	0.94	0.94	0.94	0.94
**P**	0.94	0.90	0.93	0.92	0.93	0.93	0.94	0.95	0.91	0.94	0.93	0.93	0.95	0.93	0.93	0.92	0.94	0.95	0.94	0.94
**Q**	0.94	0.92	0.92	0.93	0.94	0.94	0.95	0.95	0.94	0.95	0.94	0.93	0.93	0.93	0.94	0.93	0.94	0.95	0.93	0.95
**R**	0.93	0.90	0.93	0.93	0.94	0.93	0.93	0.94	0.92	0.94	0.93	0.93	0.93	0.93	0.93	0.93	0.93	0.94	0.93	0.94
**S**	0.93	0.91	0.93	0.93	0.94	0.94	0.94	0.94	0.93	0.94	0.93	0.93	0.93	0.93	0.93	0.93	0.94	0.94	0.93	0.94
**T**	0.95	0.92	0.93	0.95	0.96	0.95	0.96	0.95	0.95	0.96	0.95	0.95	0.95	0.92	0.96	0.94	0.95	0.95	0.96	0.96
**V**	0.95	0.92	0.94	0.94	0.95	0.95	0.96	0.95	0.95	0.95	0.95	0.95	0.95	0.94	0.95	0.94	0.95	0.95	0.95	0.95
**W**	0.94	0.92	0.94	0.93	0.96	0.94	0.93	0.94	0.93	0.95	0.95	0.94	0.94	0.93	0.94	0.94	0.94	0.94	0.94	0.95
**Y**	0.93	0.91	0.93	0.93	0.94	0.94	0.93	0.93	0.93	0.94	0.94	0.94	0.93	0.93	0.94	0.93	0.94	0.93	0.94	0.94

High ipTM
ipTM (AtLEGβ + peptide)
Low ipTM

Supplementary Figure 7. Substrate specificity is relatively broad on the non-prime side of *Arabidopsis thaliana* legumain β (AtLEGβ). Results of the AlphaFold 3-based *in silico* substrate specificity screening using the sequence of AtLEGβ and the XXXN-CL peptide are shown. To enable two-dimensional visualization, either the P2 position was fixed as alanine **(A)**

or the P4 position was fixed as alanine **(B)**. Heatmaps are colored according to the ipTM score of the respective legumain-substrate complexes.
